# Using the International Pandemic Instrument to Revitalize the Innovation Ecosystem for Antimicrobial R&D

**DOI:** 10.1017/jme.2022.79

**Published:** 2022

**Authors:** Andrea Morales Caceres, Kshitij Kumar Singh, Timo Minssen, Susan Rogers Van Katwyk, Steven J. Hoffman

**Affiliations:** 1: YORK UNIVERSITY, TORONTO, ONTARIO, CANADA; 2: UNIVERSITY OF DELHI; 3: UNIVERSITY OF COPENHAGEN, COPENHAGEN, DENMARK

**Keywords:** Antimicrobial Resistance, Pandemic Instrument, Research and Development, Innovation, Pharmaceuticals

## Abstract

The inclusion of antimicrobial resistance (AMR) and increased research and development (R&D) capabilities in the most recent outline of the World Health Organization’s (WHO’s) international pandemic instrument signals an opportunity to reshape pharmaceutical R&D system in favour of antimicrobial product development. This article explains why the current innovation ecosystem has disadvantaged the creation of antimicrobial products for human use. It also highlights how the COVID-19 pandemic experience can inform and stimulate international cooperation to implement innovative R&D incentives to bring new, life-saving antimicrobial products to the market.

## 1. Introduction

Responding to the negative consequences caused by the COVID-19 pandemic, countries around the world have initiated negotiations to create an international instrument to strengthen pandemic prevention, preparedness, and response.^1^ The latest outline of this international pandemic instrument includes antimicrobial resistance (AMR) within its scope, including various references to increasing research and development (R&D) mechanisms to address future pandemics.^2^ The inclusion of this feature is not only a welcome addition to better mitigate the threat of future global health crises. It also presents an opportunity to revamp the current pharmaceutical R&D system, which has historically disfavored antimicrobial innovation. This paper therefore argues that the World Health Organization’s (WHO’s) upcoming international pandemic instrument presents a unique opportunity to support stronger R&D mechanisms for antimicrobials in its framework. Our analysis focuses on some of the promising R&D incentive models that have been previously proposed to increase innovation for antimicrobial products for human use. Specifically, we call attention to how they could be incorporated into an international pandemic instrument given the fast pace of collaborative medical advancements during the COVID-19 pandemic. With 1.27 million deaths directly attributable to bacterial AMR in 2019,^3^ countries must grasp the opportunity presented by this international instrument to promote innovation for new — and potentially lifesaving — antimicrobials.

## 2. How the Current R&D Ecosystem Disfavors Antimicrobial Development

Despite worldwide attention given to addressing AMR in recent years,^4^ the development of new antimicrobial products has not kept up with the pace of resistance.^5^ No new classes of antibiotics have been approved since the late 1980s,^6^ and no new antibiotic class to fight Gram-negative bacteria — which are less vulnerable to antibiotics — has been approved in more than 50 years.^7^ In the last few decades, private investment has shifted towards profitable medications for noncommunicable diseases (e.g., cancer and lifestyle medications), and away from the notoriously unprofitable antimicrobial market.^8^ This shift and lack of innovation can be explained by the ‘one size-fits all’ incentives approach within the (bio)-pharmaceutical patent system, which largely disincentivizes financial investment for antimicrobial development.^9^ The financial risk that comes with developing antimicrobials in the current pharmaceutical R&D system has caused major financial actors to turn away from antimicrobial development. Most of the big pharmaceutical companies have abandoned their antimicrobial R&D programs,^10^ with companies like Novartis, Sanofi, GSK, and AstraZeneca exiting this field from 2016 to 2019.^11^ This has left small and medium-sized enterprises (SMEs) to carry the bulk of the antimicrobial R&D work, with these types of companies accounting for 81% of all antibacterial programmes in preclinical development.^12^ Unfortunately, many of these SMEs have struggled to finance their work in the later phase of drug development,^13^ leading to bankruptcies in the 2019 cases of the pharmaceutical manufacturers Achaogen and Melinta Therapeutics.^14^ This is especially concerning since it is difficult to bring back antimicrobial researchers once they are lost to other more profitable areas of biomedical research.^15^

In the last few decades, private investment has shifted towards profitable medications for noncommunicable diseases (e.g., cancer and lifestyle medications), and away from the notoriously unprofitable antimicrobial market. This shift and lack of innovation can be explained by the ‘one size-fits all’ incentives approach within the (bio)-pharmaceutical patent system, which largely disincentivizes financial investment for antimicrobial development.

Experts have highlighted two main challenges associated with bringing new antimicrobial products to the market.^16^ The first challenge — and perhaps the biggest determinant — is the small profit that antimicrobial products yield in comparison to other drugs.^17^ Not only is it expensive to develop new antimicrobials, but they also produce little financial returns. Multiple factors contribute to this dilemma, such as their brief duration of use, health regulators preferring the prescription of generic brands over patented antimicrobial products, and reimbursement schemes encouraging the sale of the cheapest drug available.^18^ Counterfeit, substandard, and falsified antimicrobial products further contribute to the market failures riddled within the antimicrobial R&D field.^19^ Additionally, the interlinked nature of antimicrobials and AMR plays a role in disincentivizing investment. The risk of an antimicrobial product being rendered useless soon after its release due to emerging resistance may also discourage investment,^20^ as does the stewardship and conservation effort to limit the use of newer antimicrobials and to treat them as drugs of last resort.^21^

The second challenge concerns the creation of new antimicrobial products, which — like the development of most new drugs — is difficult.^22^ Along with anticancer drugs, antimicrobials are one of only two classes of drugs used to kill living organisms, making it a challenge to find a treatment that will be toxic to bacteria but not the patient.^23^ Clinical drug development in this area has a low success rate in general: antimicrobial products that enter phase 1 clinical trials have approximately a 1 in 5 chance of receiving regulatory approval.^24^ While the development of new antimicrobial products is often not financially rewarding, they still carry crucial societal benefits as the last line of defence against terrible diseases. Hence, their significance should be instilled within the current (bio)pharmaceutical innovation system. Recognizing the importance of antimicrobial product development will be vital to prevent the devastating global health challenges brought on by AMR, not unlike the consequences of a global pandemic.

## 3. R&D Lessons from the COVID-19 Pandemic

After the emergence of COVID-19, the importance of global-level R&D incentives has been recognized by multilateral organizations as an essential component for future pandemic preparedness. Following the news of an upcoming international pandemic instrument, an article from the European Council outlined the potential to include better insights into R&D for pandemic solutions into this instrument, in addition to highlighting the need to share R&D solutions among nations.^25^ The 2021 Declaration from G20 health ministers also singled out R&D a “central pillar’” of pandemic preparedness, and the document stated the need for R&D to not only include new tools, but to also deliver on previous global health commitments, including the need to address AMR.^26^ Similarly, Lake et al. have expanded on the idea that R&D financing strategies could simultaneously respond to pandemic threats caused by zoonotic diseases, including AMR.^27^

Over the past two years, countries have borne witness to the lessons — both positive and negative — stemming from the COVID-19 pandemic. On an optimistic note, the accelerated pace of medical innovation during this period has demonstrated that it is possible to quickly create, license, and distribute lifesaving medical products. Few experts anticipated having a COVID-19 vaccine before the summer of 2021, especially given the lack of treatment or vaccines for other coronaviruses prior to 2020.^28^ Research and development strategies such as fast-tracking clinical processes, advanced funding, guaranteed procurement prior to approval, and cross-sector partnerships were key in the development of these life-saving medical solutions.^29^ This demonstrated that a great need and political will, may unite nations to re-shape R&D tools. Unfortunately, we have also seen how the same tools used to stimulate medical innovations — namely intellectual property (IP) rights — can exacerbate global inequities during a public health crisis. Although COVAX aimed to ensure the equitable distribution of COVID-19 vaccines in low- and middle-income countries (LMICs), the initiative’s shortcomings in global allocation and funding have been criticized.^30^ Similarly, the lack of support among World Trade Organization (WTO) members to waive the Trade-Related Aspects of Intellectual Property Rights (TRIPS) demonstrates the lack of global consensus on IP rights.^31^ During the initial vaccine rollout, these proposed solutions failed to ensure global health equity: COVAX delivered less than half of its planned two billion doses in 2021 and the TRIPS waiver has been in deadlock for more than a year since it was first tabled in October 2020.^32^ These COVID-19 lessons will be important in re-shaping R&D mechanisms to address future global health crises, especially considering the position of LMICs. Although AMR is a threat to all regions, it poses the biggest threat to these countries.^33^

## 4. Recalibrating R&D Incentives for Antimicrobials

Several commentators have elaborated on some of the existing mechanisms that could be used to stimulate the development of new antimicrobial products. Strategies to incentivize antimicrobial development are often categorized into what authors refer to as ‘push’ or ‘pull’ incentives,^34^ with numerous sub-models existing within these two incentive types. Push incentives focus on supporting the development of new antimicrobial products by mitigating financial risks and seeking to make drug development more financially appealing to investors.^35^ Examples of push incentives include research funding, tax incentives, and public private partnerships (PPPs).^36^ Conversely, pull incentives aim to bring in investors to the antimicrobial development market through outcome-based rewards.^37^ Market entry rewards, delinkage models, and patent buyouts are some of the often-cited examples of pull incentives for antimicrobial R&D.^38^

It is important to note that many of these proposed solutions remain largely untested,^39^ and will need to be evaluated as they are implemented in different contexts. Moreover, new innovation models will also have to be well-integrated into the delicate — and often conflicting — balance of antimicrobial access, conservation, and innovation,^40^ which will require societal innovation and global coordination. Acknowledging these challenges, this section will spotlight some of the promising models for antimicrobial R&D and elaborate on how these structures could be incorporated into the international pandemic instrument.

### 4.1. Delinkage: The Antimicrobial Subscription Model

One of the more promising pull incentives for increased antimicrobial R&D is the delinkage model, where a pharmaceutical company’s income is separated or ‘delinked’ from the number of antimicrobial products it sells.^41^ This subscription model guarantees a level of income well above one that pharmaceutical companies could attain from regular sales.^42^ A delinked subscription model for antimicrobial drug development is currently being piloted in the United Kingdom, where the country purchases two innovate antibiotics per year, regardless if they are used.^43^ Similar antimicrobial subscription models are being tested in Sweden.^44^

Delinkage models have been advocated by NGOs and international organizations as a strategy to not only increase R&D, but also as a potential mechanism to ensure equitable global access to antimicrobials.^45^ Given that delinkage has been on the international relations agenda of the UN High Level Panel on Access to Medicines,^46^ implementing this model through an international pandemic instrument would not be completely out of scope. In fact, the advanced financial commitments that were given to manufacture the COVID-19 vaccines mimic the delinked antimicrobial models that are currently being tested at the national level.^47^ Nevertheless, it is vital to recognize that a delinked model will require political and financial commitments — albeit smaller in scale for the latter – that are comparable to those that were provided during the COVID-19 pandemic.^48^ In order to avoid exacerbating global health inequities, a global delinkage or subscription model could ensure antimicrobial access in LMICs through other commitments that are not financial in nature. For instance, Outterson et al. propose that LMICs that are unable to make a financial contribution to the model could instead put forth other types of commitments to ensure antimicrobial effectiveness, such as conservation and surveillance efforts.^49^ Moreover, by integrating a delinked R&D model for antimicrobial products within the international pandemic instrument as a preventative measure, countries will be better prepared for future health crises, while avoiding the exacerbation of global health inequities. One of the challenges of COVAX — despite it being a good idea in principle — was the improvised nature of the arrangement, which led to a delay in country buy-in and fundraising.^50^

### 4.2. An Open-Source R&D Approach

Klug et al. argue that the inherent secrecy of pharmaceutical R&D among private companies hinders effective antimicrobial product development due to factors such as a lack of coordination.^51^ This is why a transparent, open approach to antimicrobial R&D has been advocated by these authors.^52^ The open-source approach has been successful in the software development sector, and has consequently been proposed as an additional R&D strategy for creating antimicrobial products.^53^ Existing resource-sharing platforms like the Pew Trust’s SPARK platform and the Global Antibiotic Research and Development Partnership’s (GARDP) Revive initiative are already available to share the scientific knowledge from previous drug trials to antimicrobial development companies.^54^ However, it is important to value and share both the positive and the negative outcomes of antimicrobial R&D, as has been the case in the sharing of Novartis and Achaogen’s data on the SPARK platform.^55^

While it is encouraging to see ‘access and benefit sharing’ as one of the included provisions within an early outline of the international pandemic instrument,^56^ it will be important for this principle to account for the unique challenges of the antimicrobial market. An open-source approach to antimicrobial R&D will require extensive financial backing in addition to political support. Specifically, this principle should not only emphasize the importance of transparency and benefit-sharing for antimicrobial product development, but it should also include complementary push mechanisms — such as public R&D funding — to incentivize the involvement of the private sector while ensuring equitable global access to their discoveries. To avoid a repeat of the global disagreement on patents, as seen through the proposed TRIPS waiver,^57^ it will be in every nation’s best interest to reach an international consensus on transparency, benefits sharing, and equity beforehand. Fortunately, if one believes that no market exists for antimicrobial products,^58^ a coordinated open-source approach could be among the most viable and attractive R&D solutions.

### 4.3. Public-Private Partnerships (PPPs)

Public-private partnerships (PPPs) have an established history of innovating new drugs for neglected tropical diseases.^59^ Inspired by the open innovation model, PPPs in the biomedical and pharmaceutical sector exhibit different characteristics from conventional PPP models.^60^ Unlike traditional PPP models, which are built on academic and industrial support and are backed by government or other third-party funding, PPPs in the biomedical and pharmaceutical sector involve additional players, including health foundations, patient organizations, and regulatory scientists.^61^ Notable PPPs which have engaged in the R&D of antimicrobial products include GARDP, the CARB-X global accelerator and the European Union’s Innovative Medicines Initiative (IMI)’s New Drugs for Bad Bugs (ND4BB) programme.^62^

The COVID-19 experience highlights the importance of harnessing the increased capabilities of cross-border and multisectoral PPPs to ensure the future of antimicrobial R&D. For instance, Agarwal and Gaule stated that private companies were faster than public research institutions at advancing COVID-19 vaccines to the pre-clinical stage.^63^ Conversely, while private companies largely led these clinical trials, the authors found that approximately 70% of COVID-19 trials were initiated by public health institutions (universities, hospitals, etc.).^64^ In addition to their differentiated development capabilities, capitalizing on PPPs has the added bonus of distributing the burden of financing across the public and private sectors.^65^ Given the financial risk of antimicrobial product development, an international pandemic instrument would be wise to encourage the involvement of PPPs in R&D mechanisms to secure diversified future funding.

Since international legal agreements for global health are rare, it is essential to seize the opportunity to include AMR within the scope of the international pandemic instrument, and to embed the AMR challenge within plans increase R&D capabilities. Ensuring the nations are committed — both explicitly and financially — to incentivize antimicrobial innovation responsibly will require the buy-in that an international legal framework can provide. Correspondingly, embedding R&D mechanisms within the international pandemic instrument is necessary.

### 4.4. International Collaboration for Antimicrobial R&D

Since international legal agreements for global health are rare,^66^ it is essential to seize the opportunity to include AMR within the scope of the international pandemic instrument, and to embed the AMR challenge within plans increase R&D capabilities.^67^ Ensuring the nations are committed — both explicitly and financially — to incentivize antimicrobial innovation responsibly will require the buy-in that an international legal framework can provide. Correspondingly, embedding R&D mechanisms within the international pandemic instrument is necessary for the following reasons.

First, AMR is a collective action problem that cannot be constrained by national borders. Similarly, innovative discoveries do not occur in isolation. Antimicrobial R&D needs a multinational and cross-sectoral collaboration that is coordinated, sustainable, and equity-minded. Second, having a legally binding international instrument provides opportunities to revise existing legal mechanisms — such as international health regulations — and create new ones in order to address the market failures within the antimicrobial product innovation pipeline.^68^ For instance, a previous paper highlighted the potential of an international treaty to: move funds towards the later stages of the antimicrobial development pipeline as opposed to the early stages; publish a list of all AMR-related research and development needs to eliminate unnecessary duplicates; and create and coordinate the administration of a global pooled fund for new antimicrobial products.^69^ Balasegaram et al. similarly justify the use of a legal framework for antimicrobials as the necessary ‘glue’ that will enable conservation, access, and innovation to work in the long-term at the international level.^70^ Third, an international pandemic instrument can explicitly recognize the differentiated responsibility of countries to implement — and finance — these R&D mechanisms. Most of the examples of innovative antimicrobial R&D mechanisms cited in this paper have been funded by or implemented in high-income countries (HICs). Making sure that these mechanisms are extended to LMICs, while being rolled out in conjunction with other capacity-building tools (e.g., scaling up pharmaceutical manufacturing and supply) will be essential to prevent future global health inequities.^71^

## 5. Conclusion

The inclusion of AMR in the international pandemic instrument represents a commanding opportunity to mould R&D efforts in a manner that is inclusive to antimicrobial product development. To operationalize the suggested R&D models from this paper, potential solutions within the pandemic treaty should include the following: First, countries should designate or create an international entity whose sole aim is to address AMR, including innovation. This international entity should be multisectoral to foster innovation that is sustainable and cross-cutting. Second, the international pandemic instrument must favor incorporating legally binding mechanisms to enforce global R&D models. A legally binding global agreement would make countries accountable to their declared level of commitment, provide authorization for monitoring mechanisms, and give countries the opportunity to ramp up their contributions after a period of time, in addition to streamlining AMR strategies across sectors.^72^ Third, priority should be given to enacting a pooled global innovation fund to finance the suggested antimicrobial R&D models. Ensuring that this global pool is continuously funded, while considering the differentiated responsibilities between HICs’ and LMICs’ contributions to this fund will need to be clearly outlined in the international pandemic instrument to secure its success. The creation of this funding model will also allow countries to coordinate the equitable and timely access of antimicrobials to LMICs in future scenarios, avoiding potential allocation and IP rights concerns at the international level.

Revitalizing an R&D ecosystem that has disadvantaged antimicrobial drugs for decades will not be easy. Consequently, the global health community must not let the opportunity posed by the discussion of an international pandemic instrument pass them by.

## Note

This work was supported by the Canadian Institutes of Health Research [#149542], the Social Sciences & Humanities Research Council [#895-2022-1015], and the Wellcome Trust [222422/Z/21/Z]. The funding bodies were not involved in the study design, data collection, analysis, interpretation or writing.

Timo Minssen’s and Kshitij Kumar Singh’s research for this contribution was supported by a Novo Nordisk Foundation grant for a scientifically independent Collaborative Research Programme in Biomedical Innovation Law (Grant number NNF17SA0027784).

Steven J. Hoffman is the Vice-President of Corporate Data & Surveillance at the Public Health Agency of Canada (PHAC) and previously served as the Scientific Director of the Institute of Population & Public Health at the Canadian Institutes of Health Research (CIHR). The views expressed in this article are those of the authors and do not necessarily reflect those of PHAC, CIHR or the Government of Canada. Timo Minssen serves as expert advisor to WHO Digital Health & COVID-19 Law & Ethics Committees, and the Oslo Medicines Initiative. He is also a Senior Advisor at the Swedish law firm X-Officio Advokat AB, and a scientifically independent IP Advisory Board Member of the Danish life science company Chr. Hansen A/S. The academic views expressed in this article are those of the authors and do not necessarily reflect those of the WHO, X-Officio or Chr. Hansen A/S.
